# Effects of Different Multi-Year Physical Exercise Programs on Motor Skills in Preschool Children

**DOI:** 10.3390/jfmk6030074

**Published:** 2021-09-09

**Authors:** Kristian Plazibat, Josip Karuc, Tihomir Vidranski

**Affiliations:** 1 Department of Social-Humanities Sciences, University of Slavonski Brod, 35000 Slavonski Brod, Croatia; kplazibat@unisb.hr (K.P.); tihomir.vidranski@unisb.hr (T.V.); 2 Physical Activity Measurement and Surveillance Laboratory, Faculty of Kinesiology, University of Zagreb, 10000 Zagreb, Croatia; 3 Proprio Centar d.o.o., Centre for Physiotherapy and Rehabilitation, Ante Starčevića 7A, 23000 Zadar, Croatia

**Keywords:** sex differences, fundamental motor skill, motor skills, physical activity, exercise effects, pediatrics

## Abstract

Acquiring optimal motor skills in preschool children presents a key element for proper psychomotor development. Therefore, the aim of this study was to determine gender differences and the effects of a multi-year exercise program on the level of motor skills in 161 preschool children (5–6 yo). Participants were divided into one control and three experimental groups. Motor skills were assessed with the Bruininks-Oseretsky Test of Motor Proficiency (BOT-2). To determine the difference in scores for each BOT-2 test between control and experimental groups, one-way ANOVA was used for girls and boys separately, while two-way ANOVA was used to determine the difference between the genders in the overall BOT-2 score. The results indicate that a 1-year multilateral exercise program has a positive effect on the level of motor skills in preschool children. Interestingly, additional years of participation in exercise programs yielded the maintenance of acquired motor skills level. Additionally, the exercise program affected preschool girls more than boys considering both individual and composite BOT-2 scores. According to the findings of this study, the presented exercise program could have potential benefits on multilateral development of the motor skills in preschool children, which could facilitate the balance of locomotor and manipulative skills. Therefore, the integration of multilateral programs intended for preschool children could be considered for implementation within the kindergarten curriculum.

## 1. Introduction

Early childhood presents a sensible period for cognitive, emotional and motor development [1]. Therefore, considering the emotional, psychological, social and motor aspects of children from an early age is of essential importance. Consistent physical exercise and play in childhood are considered as key components for establishing optimal growth, healthy habits, cognitive and motor skills [2]. In addition, evidence shows that the development of fundamental movement skills (FMS) in preschool children represents a key factor for optimal development of motor coordination, motor control and balance [3]. FMS are a specific set of movement skills that involve different body parts such as feet, legs, trunk, head, arms and hands, which are essential for movement stability, locomotion and manipulation [4]. According to several studies, FMS such as running, jumping, kicking, throwing and catching serve as a ‘fundamental pillar’ for success in cognitive [5], physical and sport skills [6]. For the aforementioned reasons, the development of FMS through early childhood should be emphasized in formal and informal education.

A period between two and seven years of age is considered as the period of the significant development of the FMS. This period is divided into three developmental stages: initial, basic and mature [7,8]. According to this categorization, children from the age of 6 belong to the mature phase, which is characterized by the integration of all previously learned motor components into a coordinated, accurate and efficient movement operation. Children’s movements and experiences at preschool age provide a key platform for the development of new motor skills, namely: locomotor skills (jumping and running), object-control skills (throwing and catching) and stabilizing skills (static and dynamic balance) [9]. Evidence suggests that proper motor skills development in preschool children could contribute to the maintenance of optimal body weight [10]. Consequently, the level of motor skills could possibly affect the child’s health status. A number of authors [11,12,13] point out that FMS are considered to be the foundations for the performance of more complex movement skills. In addition, Stodden et al. [14] suggested that a higher level of motor skills enables more successful participation in various physical activities. A key element of proper FMS development is a targeted physical exercise program at sensible age of maturation [13]. Indeed, it has been shown that physical exercise can facilitate the optimal development of FMS [15,16].

Still, to the best of the authors’ knowledge, none of the studies investigated the effect of multi-year physical exercise programs on children’s motor skills level. Therefore, this study aimed to determine the effect of the multi-year physical exercise program on the children’s motor skills level. Additionally, this study will explore whether this program equally affects boys and girls considering the motor skills levels.

## 2. Materials and Methods

**Figure 1:**
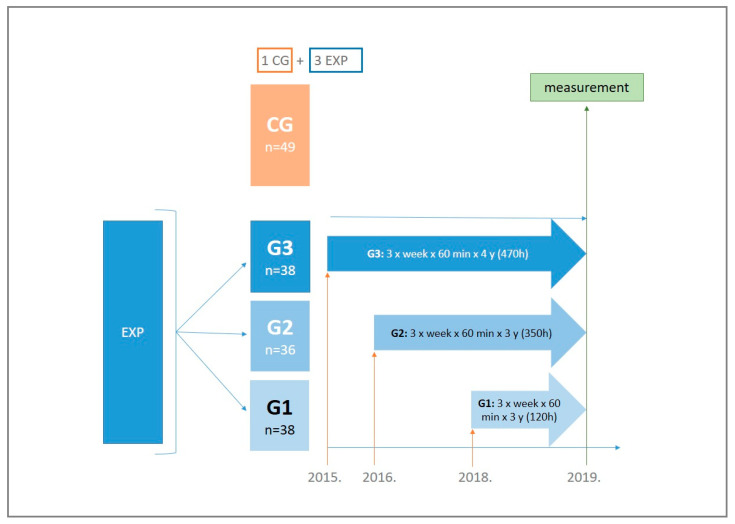
Study progress and inclusion of individual experimental groups in different time periods from 2015 to 2019. CG—control group, EG—experimental group, G1—120 h of exercise, G2—350 h of exercise, G3—470 h of exercise.

This is a cross-sectional study that included preschool children from a kindergarten in the city of Zagreb, Republic of Croatia. A total of 161 children participated (85 girls and 76 boys; total sample: 5.8 ± 0.7 years). The total sample was divided into four groups: three experimental and one control group. The experimental groups participated differently in exercise program in terms of time duration: group one (G1) exercised for 1 year (n = 38); group two (G2) exercised for 3 years (n = 36); and group three (G3) exercised for 4 years (n = 38). Participants from the control group (CG) participated in the standard preschool educational program (n = 49). This educational program was organized in the same kindergarten according to the State Pedagogical Standard of Preschool Education. All children from experimental groups were tracked and involved in the program. However, only final measurements were taken into consideration for this study. Because the program ended at the same time for each group, this study examined the difference between groups by the end of exercise programs. Consequently, all measurements were taken at the same time point in 2019 (see Figure 1).

In addition, only healthy children were included in the study, and out of the total sample of 170 children, 9 subjects dropped out. Thus, the total number of participants was 161. This study was completed according to the declaration of Helsinki, and the Ethical Committee of the Institution verified that this investigation complied with all ethical standards for scientific investigations involving human participants (class: 601-01/20-01/24; number: 251-580-15-20-02).

Body height was measured with an anthropometer where the participants stood barefoot on a flat surface, in an upright position, with relaxed shoulders and joined heels. Bodyweight was measured with a medical scale where participants stood barefoot in an upright position. To estimate the weight status, the formula body weight/body height^2^ (kg/m^2^) was used, and BMI was calculated accordingly. Bruininks-Oseretsky Test of Motor Proficiency (BOT-2) [17] was used to assess children’s motor skills. BOT-2 is the test that is suited for children from 4 up to 21 years of age and measures motor precision, motor integration, ambidexterity, manual coordination, balance, bilateral coordination, speed, agility and strength. The final test result is a standardized overall motor score, which was calculated according to the literature [18].

The physical exercise program in the kindergarten has been implemented since 2015/2016. The experimental groups were actively involved in the physical exercise program, and they were included in the program as follows: G3 starting from 2015/2016, G2 from 2016/2017 and G1 from 2018/2019. One pedagogical year was defined as the period of 10 months and included the physical exercise program of 40 weeks, containing 120 training units (hours). This means that when the measurement took place, the total number of hours in the physical exercise program for G1 was 120, followed by the G2 with 350 h and the G3 with 470 h. The exercise program was organized three times per week (three sessions) with a duration of 60 min per each session, while two physical education teachers were responsible for the realization, control and assigning of the workload for the children. Two physical education teachers systematically planned, programmed and conducted physical exercises with the children. Specific training sessions were carried out so that everyone followed the same protocol. The physical exercises program was focused on the multilateral psychophysical development of the child. Additionally, within the physical exercises program, a schedule with the distribution of the workload through program contents was defined. Within one school year, the participants were introduced to basic gymnastics exercises, various jumps and runs, polygon obstacles, manipulation of various props and learned the basics of team sports such as football, handball, basketball and volleyball (Table 1). During the implementation of the physical exercises program, the primary task was to facilitate the development of the child’s positive life habits and the development of cognitive and motor skills. Children’s diets had been planned according to the recommended amounts of energy to be consumed in a day, in accordance with the daily workload. This was completed in accordance with the provisions of the kindergarten teacher (Table 1).

First, descriptive analysis for basic anthropometric variables was completed for girls and boys separately. Second, to determine difference in scores for each BOT-2 test between control and experimental groups, one-way ANOVA was used for girls and boys separately. To investigate difference between the gender in the overall BOT score, two-way ANOVA was employed, using gender and years of experience as fixed factors. The level of the statistical significance was set at *p* < 0.05.

## 3. Results

Body height, body weight and BMI averaged (±SD) 117 ± 6.2 cm, 21.8 ± 4.1 kg and 15.8 ± 2.1 kg/m^2^ for the total sample, respectively. A two-way ANOVA did not reveal a significant interaction between gender and the years of exercise (*p* = 0.48). However, analysis has shown a statistically significant difference in the overall BOT-2 test score among both girls and boys between most groups (see Table 2).

In girls, a one-way ANOVA has shown a statistically significant difference between CG and G1, CG and G2 and between CG and G3 in the following variables: fine motor precision (*p* = 0.0004–0.0001), ambidexterity (*p* < 0.0001), hand coordination (*p* = 0.01–0.0003), balance (*p* = 0.001–0.0009) and strength (*p* < 0.0001).

**Figure 2:**
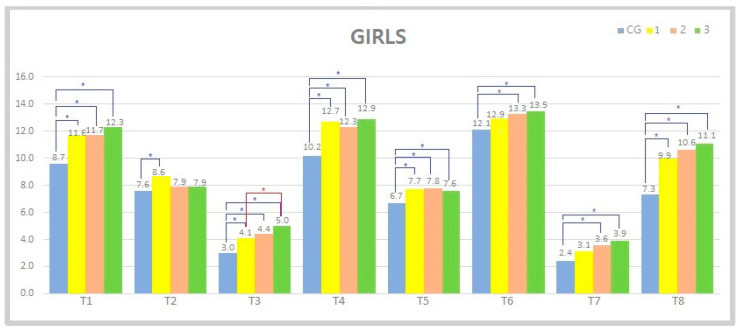
Representation of variables and mean values within individual groups in girls. (CG: control group, 1: experimental group that practiced for 120 h, G2—350 h of exercise, G3—470 h of exercise). (T1—motor precision, T2—motor integration, T3—ambidexterity, T4—hand coordination, T5—balance, T6—bilateral coordination, T7—speed and agility, T8—strength); * represents a statistically significant difference between the groups (*p* < 0.05).

In other variables, statistical significance between control and other experimental groups was not obtained among all groups. For detailed results, see Figure 2.

**Figure 3:**
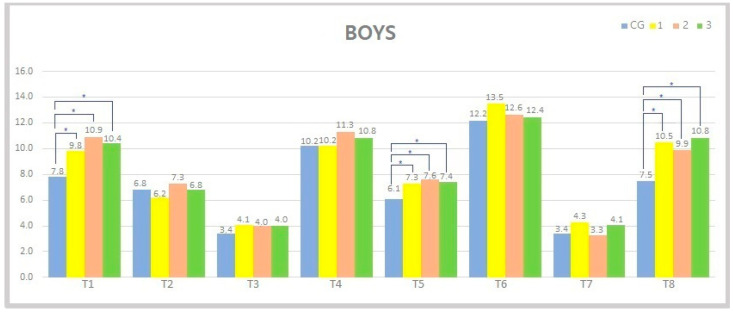
Presentation of variables and mean values within individual groups in boys (CG—control group, EG—experimental groups, G1—120 h of exercise, G2—350 h of exercise, G3—470 h of exercise). (T1—motor precision, T2—motor integration, T3—ambidexterity, T4—hand coordination, T5—balance, T6—bilateral coordination, T7—speed and agility, T8—strength); * represents a statistically significant difference between the groups (*p* < 0.05).

Among boys, a significant difference between CG and G1, CG and G2 and CG and G3 was observed in three variables (fine motor precision: *p* = 0.009–0.0003; balance: *p* = 0.01–0.0005 and strength: *p* = 0.001–0.0002). However, no significant difference was evident in other variables between CG and other experimental groups (see Figure 3).

## 4. Discussion

The results of this study indicate that a 1-year multilateral physical exercise program has a positive effect on the level of motor skills in preschool children. Moreover, our results show that additional years of participation in exercise programs yielded the maintenance of the acquired FMS levels. Additionally, evident sex difference was observed in the effects of exercise programs on FMS level. More specifically, the findings of this study suggest that the program affected preschool girls more than boys considering both individual and composite BOT-2 scores.

Regarding sexual dimorphism, differences were found between CG and experimental groups in all eight components of movement skill among girls, while among boys, these differences were evident in only three tests (see Figure 2 and Figure 3). The effect of a 1-year additional exercise program on FMS was evident in both sexes (differences between CG and experimental groups in BOT-2 score were 56 and 64 for boys and girls, respectively). However, additional 3- and 4-year participation in exercise programs did not significantly improve FMS among children. It is important to note that the aforementioned finding does not imply that children do not need to participate in additional programs for more than 1 year. Moreover, this kind of program should be emphasized through the period of early childhood in order to maintain the acquired level of FMS that was gained in the first year of the program. The authors of this study suggest that this could potentially reduce deterioration of motor development and the development of suboptimal motor skills. However, more studies are needed in order to investigate the aforementioned effects on motor development among the different pediatric populations. Our results suggest that children from the control group who participated in the standard preschool educational program have considerably lower results in motor skill tests. Therefore, looking from the practical standpoint, this kind of program should be enriched with multilateral physical exercises interventions in order to acquire optimal motor skill development. Overall, the presented exercise program has resulted in positive benefits in preschool children compared to the standard preschool educational program.

Although this program affected both sexes, one question still remains: *‘Why did the additional years of exercise program not result in more significant effects on FMS level in preschool children?’* The explanation for such findings in the current study may be due to the fact that the participants did not exercise daily. Consequently, it would be necessary to increase the weekly frequency of physical activity, which could potentially have an effect on FMS level. This approach is in accordance with the recommendations of several scientific institutions [19,20], which suggest that children should accumulate at least 180 min of physical education through preschool education activities each day, while the guidelines point to 11,500 steps daily [21]. However, in this study, we did not control for physical activity level, and the above-offered potential explanations should be taken with caution. Another possible reason for these findings could be due to stabilization of learned movement skills after 1 year of practice, where any additional year spent practicing would not yield an improvement of movement skill. However, the authors point out that maintaining the acquired level of FMS through all years of kindergarten education should be a priority in the population of preschool children. Still, more researches are needed in order to explore the aforementioned effect on movement skills in preschool children.

The results of this study differ from the results of a more recent study [22] that included a sample of 31 participants (5.59 ± 0.77 yo). The results of Šalaj et al. [22] showed that there were no sex differences in gross motor quotient and locomotor or manipulative skills among preschool girls. However, differences were found in gross motor quotient and locomotor skills when comparing selected with the non-selected gymnast girls. Additionally, our overall BOT-2 test results differ from the results in the study completed by Kezić et al. [23] (n = 70, 6.0 ± 0.5 years), where participants showed lower results in the following tests: hand coordination (1 to 12.00), bilateral coordination (2 to 7.00) and strength (0 to 8).

Furthermore, Guo et al. [24] found a significant correlation between the effectiveness of motor skill and physical activity (r = 0.14–0.17) where they stressed that early interventions of programmed physical activity are crucial for the development of FMS. Although these studies examined children of the same chronological age, the results of our study differ from the results of the above-mentioned studies. This could be probably because the aforementioned studies had a differently oriented concept of exercise programs. Additionally, the results of the above-mentioned researches show a lower level of locomotor and manipulative skills. Although the results of this study are different from the aforementioned researches, this study gave new insights into the impact of various exercise programs on the motor development of children.

### Strengths and Limitations

This study has three advantages. First, this study included three experimental groups with different previous motor experiences and different exercise volumes, which can allow better conclusions about the effects of the exercise program on the measured motor skill parameters. Second, this study followed children for 4 years through a sensitive period of development. Lastly, given the long duration of the physical exercise program, a relatively large number of participants remained throughout the study, which contributes significantly to the quality of this study. However, this study has several limitations. In this study, we did not control for other variables such as children’s cognitive status, family’s socioeconomic indicators and parents’ physical activity. Additionally, physical activity of the children was not measured outside of the school environment. This could potentially affect the results and conclusions drawn. Additionally, this research has a cross-sectional design, which limits causal conclusions. This study did not include pretest measurements and did not control for the above-mentioned variables, and therefore, these limitations need to be considered while interpreting the results of this research. Lastly, the sample is not representative, which may influence the generalizability of the results in the context of the general preschool children population.

## 5. Conclusions

According to the results of our study, authors support that preschool children take up regular exercises with the multilaterally directed exercise program as covered within this study. In order to increase the effectiveness of motor skills, further researches are needed regarding the validation of new methods and the construction of multilateral exercise programs intended for preschool children. Conclusively, the presented exercise program could have potential benefits on multilateral development of the motor skills in preschool children, which could facilitate the balance of locomotor and manipulative skills. Therefore, the integration of multilateral programs intended for preschool children could be considered for implementation within the kindergarten curriculum.

## Figures and Tables

**Table 1:** Microcycle: example of the program content for three lessons per week.

Introductory	Preparatory	Main Part of the Lesson	Game	Final Part of the Lesson
*a*				
Running with different tasks	General preparatory exercises with sticks	1. Dribbling in place basketball (B); 2. Throwing the ball into the hoop	Game: relay play by dribbling	Game: fly, fly
*b*				
Running in a circle	General preparatory exercises with balls	Front tuck mount and dismount (vaulting box)	Relay game on the one leg	Game: guess who I am
*c*				
Running in pairs	General preparatory exercises in pairs	1. Passing and catching the ball (B); 2. Obstacle polygon	Game: dodgeball	Game: cone shooting with the ball

Note: *a*, *b*, *c*: example of the program content for first (a), second (b) and third (c) lesson per week.

**Table 2:** Two-way ANOVA results for the overall BOT score within and between groups of different years of experience and gender.

Group				
	CG	G1	G2	G3
**Boys**	53.88	62.67 ^1^	62.58 ^2^	62.94 ^3^
**Girls**	54.88 ^4,5,6^	64.74 ^7,8^	66.35 ^9,10^	68.36 ^11,12^

Note: CG: control group; G1: group of participants that were included in the 1-year exercise program; G2: group of participants that were included in the 3-year exercise program; G3: Group of participants that were included in the 4-year exercise program; ^1^ difference between CG boys and G1 boys (*p* = 0.002); ^2^ difference between CG boys G2 and boys (*p* = 0.0008); ^3^ difference between CG boys and G3 boys (*p* = 0.001); ^4,5,6^ difference between CG girls and G1 boys (*p* = 0.01), G2 boys (*p* = 0.97), G3 boys (*p* = 0.99); ^7^ difference between CG boys and G1 girls (*p* = 0.00003); ^8^ difference between CG girls and G1 girls (*p* = 0.00005); ^9^ difference between CG boys and G2 girls (*p* = 0.00003); ^10^ difference between G1 girls and G2 girls (*p* = 0.99);^11^ difference between CG boys and G3 girls (*p* = 0.00003); ^12^ difference between CG girls and G2 girls (*p* = 0.00004).

## Data Availability

Individual participant data will be available and will be shared along with additional documents (all of the individual participant data with consent form and analysis plan). Data will be available immediately following the publication with no end date to anyone who wishes to access the data (for any types of analyses). Data will be available on request to the corresponding author.
